# Effect of cholecystectomy on hepatic fat accumulation and insulin resistance in non-obese Hispanic patients: a pilot study

**DOI:** 10.1186/s12944-017-0525-3

**Published:** 2017-06-30

**Authors:** Víctor Cortés, Nicolás Quezada, Sergio Uribe, Marco Arrese, Flavio Nervi

**Affiliations:** 1 Departamento de Gastroenterología, Facultad de Medicina, Pontificia Universidad Católica de Chile, 6513677 Santiago, Chile; 2 Departamento de Nutrición, Facultad de Medicina, Pontificia Universidad Católica de Chile, 6513677 Santiago, Chile; 3 Departamento de Cirugía Digestiva, Facultad de Medicina, Pontificia Universidad Católica de Chile, 6513677 Santiago, Chile; 4 División de Imágenes, Laboratorios y Patologías, Departamento de Radiología y Centro de Imágenes Biomédicas, Facultad de Medicina, Pontificia Universidad Católica de Chile, 6513677 Santiago, Chile; 5 Centro de Envejecimiento y Regeneración (CARE), Departamento de Biología Celular y Molecular, Facultad de Ciencias Biológicas, Pontificia Universidad Católica de Chile, Santiago, Chile

**Keywords:** Gallbladder, Cholecystectomy, Steatosis, NAFLD, Insulin resistance

## Abstract

**Background:**

Nonalcoholic fatty liver disease (NAFLD) is highly prevalent worldwide. Experimental studies have shown that cholecystectomy (XGB) increases hepatic fat content in mice and appears associated to NAFLD in large retrospective population-based studies. The aim of this study was to prospectively assess the effects of XGB on hepatic fat content (HFC) and insulin resistance (IR) in non-obese, middle aged Hispanic subjects.

**Methods:**

Twenty-six gallstone patients undergoing elective XGB and 16 control subjects with normal livers and gallbladders at ultrasonography were prospectively followed 24 months for changes in HFC and IR. Clinical, biochemical determinations and hepatic imaging were performed at baseline and 24 months after surgery. MRI technique quantified HFC in four hepatic segments. IR was assessed by the Homeostasis Model Assessment (HOMA_-IR_) index.

**Results:**

Initial body mass index (BMI) was 25.6 ± 0.4 and 24.3 ± 1.0 in the control and XGB groups of subjects, respectively. Serum insulin level increased from 8.1 ± 0.7 to 10.0 ± 1.9 (μU/ml) 24 months after surgery in XGB patients (*p* < 0.05); no significant changes were detected in control individuals. Median HOMA_-IR_ index increased from 1.31 (interquartile range, 1.01-1.68) to 2.20 (interquartile range, 1.57 - 2.60) 24 months after XGB, (*p* < 0.003). Median HOMA_-IR_ index of control subjects remained unchanged at the end of the study. Serum apoB concentration increased from 61.5 ± 3.4 to 79.0 ± 7.8 (μg/ml) in XGB patients (*p* < 0.03). Serum apoB levels remained within normal ranges in both periods of the study in control subjects. HFC significantly increased in 2 of the 4 segments 24 months after XGB: right posterior hepatic lobe (from 5.3 ± 0.2% to 6.0 ± 0.2%, *p* > 0.04) and right anterior hepatic lobe (from 5.8 ± 0.2% to 6.6 ± 0.3%, *p* < 0.02). The average HFC of the four hepatic segments studied slightly increased from 5.4 ± 0.2 to 5.8 ± 0.3 2 years after XGB (*p* < 0.03). No significant changes were found in HFC in the control subjects at the end of the study.

**Conclusions:**

Elective XGB increases HFC, HOMA_-IR_ index and serum apoB concentration. These results support the notion that XGB is a risk factor non-alcoholic fatty liver disease and other IR – associated disease conditions.

## Background

Nonalcoholic fatty liver disease (NAFLD) is highly prevalent worldwide with an estimated rate of 30% among the adult population diagnosed by abdominal ultrasound [1–3]. South American and Mexican American Hispanics have the highest prevalence rates of NAFLD with figures ranging between 30 to 50% of the population [4]. NAFLD is frequently associated to obesity, type 2 diabetes, atherosclerosis and cholesterol gallstones [5–7] with these entities sharing some metabolic alterations. NAFLD has a complex bidirectional pathogenic interrelationship with the metabolic syndrome (MS). NAFLD is considered both, a metabolic manifestation of MS and in some cases, a primary determinant of insulin resistance (IR) and the metabolic abnormalities clustered in the MS [8–12]. The natural history and possible evolution of NAFLD varies greatly in seriousness, starting as an accumulation of fat in the hepatocyte and potentially evolving towards nonalcoholic steatohepatitis (NASH), cirrhosis and hepatocellular carcinoma [13–15].

The cause of the increase of triglycerides (TG) in NAFLD has not been completely unveiled. However, it is known that IR is a fundamental pathogenic factor that alter each of the processes that regulate TG concentration in the liver by increasing hepatic lipogenesis and lipolysis from adipocytes, and decreasing peripheral lipoprotein lipase activity, producing an increase of chylomicron and VLDL remnants, which are rapidly cleared by the liver, thus resulting in a net TG accumulation in the organ [16–18].

Cholecystectomy (XGB) is the recommended treatment for gallbladder (GB) diseases, including gallstone disease (GSD) and cholecystitis [19]. Indeed, XGB is one of the most commonly performed surgical procedures worldwide [20, 21] and is considered a low-risk surgical procedure with no major long-term health implications. However, recent evidence shows that the GB may not only be a simple reservoir that stores, concentrates and delivers bile into the intestine for lipid absorption. Through its critical role in regulating bile acid (BA) metabolism and the recent finding that GB mucosa is rich in the hormone Fibroblast Growth Factor 19 (FGF19), this organ may have a physiological role in whole body metabolic homeostasis [22, 23]. In fact, the possibility that GB ablation may have metabolic consequences has emerged [24] due to recent retrospective epidemiological studies showing that XGB may be a risk factor of NAFLD) [25, 26] and metabolic syndrome [27]. Concordantly, experimental studies in mice have shown that XGB increases basal metabolic rate, serum and hepatic triglycerides concentration and very low-density lipoprotein (VLDL) production [28, 29]. Moreover, XGB has been associated with elevated VLDL levels [30], deteriorated postprandial glycemic control [31] and weight gain [32] in humans. The underlying mechanisms of these effects remain ill defined. Since BAs are important signaling molecules in controlling lipid and carbohydrate metabolism [33–35] it is plausible that XGB may influence whole-body metabolic regulation through changes in BA physiology and potentially contributing to the development of IR and MS - associated conditions, particularly NAFLD [19–21].

To better define the potential role of XGB as a risk factor of NAFLD, the present study aimed to prospectively evaluate the effects of XGB on liver fat content and IR, one of the most relevant factors underlying NAFLD development [36]. To that end, we determined IR by the homeostatic model assessment index (HOMA-_IR_) [37] and hepatic fat by magnetic resonance imaging (MRI) [38, 39] in a series of non-obese GSD patients over a period of 24 months after elective XGB.

## Methods

### Patient selection

This study agreed with the ethical guidelines of Declaration of Helsinki (1975) and was approved by the Institutional Review Board for Human Studies of the Faculty of Medicine at *Pontificia Universidad Católica de Chile*. All participants gave informed written consent prior to participate in this survey.

We arbitrarily included 30 patients harboring asymptomatic gallstone disease subjected to elective laparoscopic XGB at the Clinical Hospital of the *Pontificia Universidad Católica de Chile*, between January and August 2013. We also studied a group of 20 control subjects with similar clinical and biochemical characteristics and normal GBs at abdominal ultrasound. Twenty-six cholecystectomized patients and 16 control subjects completed the study 24 months later.

All subjects were 35 to 55 years old, non-diabetic and non-obese who agreed to participate in the study. Inclusion criteria were: a) body mass index (BMI) < 28 and no change in weight of more than 3 kg in the last 6 months before entering the study; b) fasting glucose, lipid levels and liver function tests within normal values; c) absence of hepatic steatosis assessed by abdominal ultrasound; d) no use of glucose- and lipid-lowering drugs, estrogens or androgens. Exclusion criteria were: a) abnormal serum alanine aminotransferase (ALT) or aspartate aminotransferase (AST) concentrations; b) hepatic pathologies on MRI; c) alcohol consumption >20 g per day in the last 3 years; e) substance abuse; f) significant systemic diseases and g) inability to undergo MRI.

### Clinical and biochemical assessments

Baseline clinical evaluation included medical history, physical exam, and height and weight measurements to calculate body mass index (BMI). Venous blood samples were obtained after 12 h of fasting. Glucose, lipids, insulin, apolipoprotein B (apoB), ALT and AST serum levels were measured at the central laboratory of *Pontificia Universidad Católica de Chile* Health Network. We used the homeostasis model assessment index (HOMA_-IR_) to define IR at fasting [20, 24]. We defined IR for non-obese middle-aged subjects when HOMA_-IR_ values were ≥2.53 according to a population –based Chilean study [40].

### Magnetic resonance imaging protocol and imaging analysis

A magnetic resonance imaging (MRI) scanner (1.5 T Philips Achieva MRI scanner, Best, The Netherlands) was used to quantitate hepatic fat content (HFC) A fat fraction map of the liver was generated based on IDEAL method [41]. Briefly, echo times were set to TE = 2.3/ 3.5/ 4.7 ms, corresponding to the optimal echo times in IDEAL algorithm for 1.5 T [42]. TRs and flip angles were chosen experimentally using fat-water-emulsion phantoms [43].

MRI data was processed with OSIRIX software (OsiriX Foundation, Geneva, Switzerland) to extract water and fat images. Afterwards, fat fraction map was extracted using image calculator tool of Image J software (NIH, Bethesda, MA, USA). In the fat fraction map, one region of interest (ROI) was set in four segments of the liver avoiding vascular structures. Four segment of the liver were analyzed: right posterior hepatic lobe (RPHL), anterior hepatic lobe (RAHL), left medium hepatic lobe (LMHL) and left lower hepatic lobe (LLHL). Mean FF was calculated for each ROI. Additionally, a mean value of the fat fraction of the liver was calculated by averaging the fat fraction values of the four ROI.

### Statistical analysis

Parametric data were presented as mean ± SE of the mean and non-parametric data were expressed as medians with interquartile values. Student’s *t* test was used to compare parametric data. Wilcoxon test was used for non-parametric variables and paired Chi-square tests were used to compare frequencies. Prism software (Version 6, http://www.graphpad.com/) was used for statistical analysis. Differences were considered significant with *P* values <0.05.

## Results

Table 1 show that age and sex distribution was similar in XGB patients and control subjects. Importantly, BMI was in the upper limit of the normal range in both groups at the beginning and end of the study. Fasting serum glucose, insulin, lipids and apoB were also equivalent and within the normal range at the beginning of the study (Table 1). Twenty-four months after XGB, serum apoB levels of control individuals remained within normal ranges at end of the study (68.1 ± 3.4 vs 63.1 ± 2.8 (μg/ml), respectively). Serum apoB concentration of cholecystectomized patients increased from 61.5 ± 3.4 to 79.0 ± 7.8 (μg/ml) in cholecystectomized patients (*p* < 0.03).

**Table 1:** Age, weight, Body Mass Index (BMI), and biochemical parameters of the control and cholecystectomized individuals, at the beginning and end of the study

Characteristic	Control (*N* = 16)		Cholecystectomy (*N* = 26)	
Time (months)	0	24	0	24
Age	38.2 ± 2.6		37.3 ± 1.7	
Female/male	10F/6M		18F/8M	
Body weight (kg)	71.5 ± 3.0	72.0 ± 3.2	67.4 ± 1.8	71.5 ± 2.3
BMI (kg/m^2^)	25.6 ± 0.4	26.5 ± 0.5	24.3 ± 1.0	24.4 ± 1.1
ALT (U/L)	23.5 ± 3.2	27.9 ± 4.7	23.5 ± 3.2	27.9 ± 4.7
AST (U/L)	23.5 ± 7.1	11.6 ± 7.7	21.4 ± 3.7	22.7 ± 3.1
Serum glucose (mg/dl)	85 ± 1.5	84 ± 1.6	79 ± 1.6	81 ± 1.5
Serum insulin (μU/ml)	8.5 ± 1.7	9.4 ± 1.3	8.1 ± 0.7	10.0 ± 1.9^*^
Total cholesterol (mg/dl)	179 ± 8	182 ± 7	172 ± 6	197 ± 8
LDL cholesterol (mg/dl)	105 ± 7	110 ± 8	100 ± 6	117 ± 7
HDL cholesterol (mg/dl)	50 ± 3	49 ± 3	48 ± 2	55 ± 2
Triglycerides (mg/dl)	118 ± 12	117 ± 11	121 ± 10	127 ± 12
Serum apoB (μg/ml)	68.1 ± 3.4	63.1 ± 2.8	61.5 ± 3.4	79.0 ± 7.0^#^

*Plus* minus values are means ± SE, *BMI* body mass index, *AST* aspartate aminotransferase, *ALT* alanine aminotransferase, *LDL* low density lipoprotein, *HDL* high density lipoprotein, *apoB* apoprotein B. ^*^ *p* < 0.05, ^#^ *p* < 0.03

### XGB is associated with elevated serum insulin concentration and HOMA_-IR_ index

**Fig. 1:**
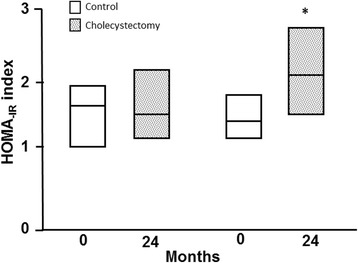
Effect of XGB on HOMA_-IR_ index. Values represent the median and the interquartile ranges. White columns represent results at the beginning of the study and columns with grey dots represent results obtained at the end of the study. *P < 0.02; ^¶^ *P* < 0.001

Serum insulin level increased from 8.1 ± 0.7 to 10.0 ± 1.9 (μU/ml) 24 month after surgery in XGB patients (*p* < 0.05), whereas no significant changes were detected in control individuals (Table 1). As shown in Fig. 1, median HOMA_-IR_ index of control subjects remained unchanged, from 1.42 (interquartile range, 1.02 – 1.86) to 1.74 (interquartile range, 1.11 - 2.39) during the observational period. Contrariwise, median HOMA_-IR_ index increased from 1.31 (interquartile range, 1.01-1.68) to 2.20 (interquartile range, 1.57 - 2.60) 24 months after XGB, (*p* < 0.003).We operationally defined IR when HOMA_-IR_ was ≥2.53 for middle-age, non-obese Chilean subjects [24]. Upon this definition, 15% of individuals in both groups had IR at the beginning of the study. Twenty-four months later, 61% of the patients subjected to XGB had HOMA_-IR_ ≥ 2.53, compared with only 12% in the control group of individuals (*p* < 0.001). Considered together, these results suggest that XGB determines increased circulating insulin levels and favors IR in non-obese individuals.

### XGB is associated with higher hepatic fat

**Fig. 2:**
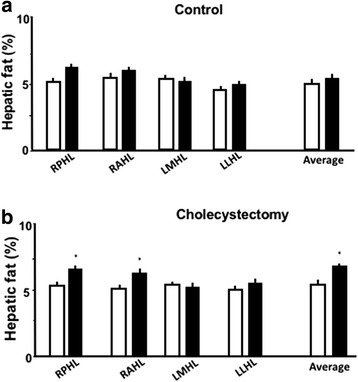
Effect of XGB on hepatic fat content. Columns represent the mean ± SE of the percentage of HFC observed at the beginning and after 24 months of follow-up in control (panel **a**) and cholecystectomized subjects (panel **b**). White and black columns represent the values obtained at the beginning and end of the study. Four liver segments were analyzed: right posterior hepatic lobe, RPHL; right anterior hepatic lobe, RAHL; left medium hepatic lobe, LMHL; left lower hepatic lobe, LLHL, and mean value of the four lobes. White columns represent values at the beginning of the study and columns with grey dots represent values obtained at the end of the study. * *P* < 0.03

At the beginning of the study, all individuals of both groups had hepatic fat (HFC) lower than 8% as assessed by MRI, indicating that they had no, or mild steatosis [21,22]. Twenty-five cholecystectomized patients and 15 control subjects had a second MRI after 24 months of follow–up. Figure 2 shows that HFC of the four segments studied remained unchanged after 24 months of follow up in the control group. Contrariwise, patients subjected to XGB significantly increased HFC in the right posterior hepatic lobe (RPHL) (5.3 ± 0.2% to 6.0 ± 0.2%, *p* > 0.04) and in the right anterior hepatic lobe (RAHL) (5.8 ± 0.2% to 6.6 ± 0.3%, *p* < 0.02). The average value of HFC of the 4 hepatic segments, slightly increased 7%, from 5.4 ± 0.2% to 5.8 ± 0.20% (*P* < 0.05) in cholecystectomized patients. No significant change was found in the control group.

## Discussion

This prospective pilot study shows that XGB is associated with a significant increase in HFC, serum apoB and insulin levels, as well as in HOMA_-IR_ index, in a cohort of non-obese Hispanic subjects. These findings provide support to the contention that, whereas XGB cures GSD and eliminates the risk of GB cancer, it may have relevant negative metabolic consequences [22, 24], contributing to the development, or worsening of IR and its consequences. Of note, IR is a major pathophysiological determinant of type 2 diabetes, atherosclerotic vascular diseases [44–46] and various cancers associated with obesity [47]. Moreover, NAFLD has become the most prevalent liver condition worldwide and has intrinsic links with IR – MS and a number of associated disease conditions, including cardiovascular diseases [9].

The mechanisms underlying the observed metabolic effects of XGB in the present study remain to be clarified. The GB could theoretically regulate whole body IR/sensitivity by direct mechanisms, through signaling factors secreted by its mucosa, or indirectly by regulating the flux mass of BAs through the enterohepatic and systemic circulation during the fast-feeding cycles. One of the GB-derived factor that play a role in this setting could be FGF19. This ileal hormone regulates BA synthesis and GB filling and is highly expressed in GB mucosa and secreted into bile [4]. FGF19 has direct systemic metabolic effects on lipid and carbohydrate metabolism [48, 49]. Of note, serum FGF19 levels decrease after XGB [50] and have been found decreased in patients with NAFLD [51, 52]. Thus, it could be hypothesized that dysregulation of FGF 19 after XGB may in part mediate the metabolic consequences of XGB observed in this study.

With regard to XGB-induced changes in BA physiology, it is generally accepted that XGB determines a decrease in the size of the BA pool [22] and increases the enterohepatic recirculation rates of BA in the fasting state [53, 54]; the BA pool recycles at least twice as often as normal after XGB [55]. BA are recognized as relevant signaling molecules [34] that may be critically involved in NAFLD development through their hepatic and extrahepatic effects regulating lipid and carbohydrate metabolic pathways, as well as energy homeostasis [56]. On these grounds, XGB may theoretically determine elevated exposure of both cell surface and nuclear receptors to BAs, leading to pathological effects on triglycerides and glucose homeostasis [22, 23, 33–35].

In our view, three major conclusions can be drawn from this study. The first relates to the observation that XGB favors lipid accumulation in the liver 24 months after surgery. We obtained these results using validated highly sensitive MRI methods [38, 39] that allowed us to evaluate the statistical significance of small changes in the fat fraction of individual liver segments. In fact, although the actual magnitude of the increase in HFC after XGB was small, it is remarkable that these changes occurred in a relatively short time period and reached statistical significance. This result is consistent with the increased prevalence of NAFLD in patients that have underwent XGB reported in two large retrospective population-based studies in North American [25] and Asian populations [26]. Furthermore, a study based on the US National Health and Nutrition Examination Survey (NHANES) showed that XGB, but not GSD, was associated to cirrhosis and elevated serum liver enzymes [57]. Secondly, in our best knowledge, this is the first prospective study showing that GB ablation associates with increased serum insulin levels and HOMA-_IR_ index in humans. These findings are consistent with a number of retrospective cross-sectional epidemiological analyses showing that cholecystectomized patients have increased risks of MS [27], arteriosclerotic vascular diseases [58] and NAFLD [25, 26]. Our third conclusion is that the observed increase in HFC and HOMA-_IR_ index 24 months after XGB supports the hypothesis that NAFLD and IR could develop after XGD due to the recently discovered metabolic roles of GB [22]. In a previous mouse study, we found that XGB increases serum and hepatic triglyceride concentrations along with higher VLDL and apoB production [28]. Although not dynamic measurements of insulin sensitivity/resistance are available for those experiments, the metabolic changes observed are suggestive of hepatic IR [45, 46]. In addition, the elevated levels of apoB, 24 months after XGB is also suggestive of systemic and hepatic IR, since insulin increases the secretion and decreases the clearance of apoB [59].

Based on our current results and the commented experimental and epidemiological evidence, it is theoretically conceivable that a primary altered GB function, even in the absence of GSD, could induce not only gallstone formation, but also potentiate systemic negative metabolic changes. In fact, diabetic patients and individuals with IR with no evidence of gallstones, have abnormal GB motility [60, 61], possibly changing their BA kinetics and exposing enterohepatic and peripheral tissues to changes in BA metabolic effects.

## Conclusions

Our results are consistent with previous retrospective epidemiological surveys showing that XGB, but not GBD appears associated as a risk factor of metabolic syndrome-associated complications, particularly to NAFLD. Although this study was performed in a relatively small number of non-obese individuals, it is plausible that XGB could further increase the risk of IR and associated disease conditions in individuals with elevated basal metabolic risk, including obesity and diabetes. Our findings stress the necessity to prevent GSD and perform longer and larger prospective studies to more precisely disclose the metabolic consequences of GB ablation.

## Abbreviations

ALT: Alanine aminotransferase
Apo B: Apolipoprotein B
AST: Aspartate aminotransferase
BA: Bile acid
BMI: Body mass index
FF: Fat fraction
FGF19: Fibroblast growth factor 19
GSD: Gallstone disease
HDL: High density lipoprotein
HFC: Hepatic fat content
HOMA-IR: Homeostasis model assessment index
IDEAL: Iterative decomposition of water and fat with echo asymmetry and least squares estimation
LDL: Low density lipoprotein
LLHL: Left lower hepatic lobe
LMHL: Left medium hepatic lobe
MRI: Magnetic resonance imaging
ms: Milliseconds
NAFLD: Non-alcoholic fatty liver disease
NASH: Non-alcoholic steatohepatitis
RAHL: Right anterior hepatic lobe
ROI: Region of interest
RPHL: Right posterior hepatic lobe
VLDL: Very low-density lipoprotein
XGB: Cholecystectomy
